# Accuracy of dietary intake assessments using food records based on photographic images captured by visually impaired people

**DOI:** 10.1371/journal.pone.0280725

**Published:** 2023-02-06

**Authors:** Thaís Lima Dias Borges, Marcos Felipe Silva de Lima, Severina Carla Vieira Cunha Lima, Ursula Viana Bagni

**Affiliations:** 1 Postgraduate Program of Nutrition, Federal University of Rio Grande do Norte, Natal, Rio Grande do Norte, Brazil; 2 Faculty of Health Sciences of Trairi, Federal University of Rio Grande do Norte, Santa Cruz, Rio Grande do Norte, Brazil; 3 Department of Nutrition, Federal University of Rio Grande do Norte, Natal, Rio Grande do Norte, Brazil; 4 Department of Social Nutrition, Fluminense Federal Nutrition, Niterói, Rio de Janeiro, Brazil; The University of Hong Kong, HONG KONG

## Abstract

Traditional methods to assess dietary intake have limited and questionable application in visually impaired people since the lack of vision and low leading role in their diet make it difficult to quantify and detail the food consumed throughout the day. Thus, this study investigated whether it is possible to accurately identify foods and estimate their quantities using food records based on photographic images captured by visually impaired people. A panel of experts composed of nutritionists (n = 20) assessed these records comprising three standardized meals (breakfast; lunch/dinner; snack) from visually impaired people (n = 40) using two different protocols (frontal photo; aerial photo). Each nutritionist reported an estimated food record for each photographic image, which was compared to its respective weighed food record. For both frontal and aerial photos, experts were frequently correct for the number of food items in the meal (95.0% or over for breakfast, 100% for lunch/dinner, and 100% for snacks). All experts identified at least 11 of the 13 food items, but the majority correctly estimated the food amount only for 23% of the items. Compared to the weighed food record, the photographic records underestimated the amount of 61.5% of food items based on frontal photos, and of 76.9% of food items based on aerial photos. While most foods could be identified by photographic images captured by visually impaired people enabling a qualitative assessment of the diet, they could not be quantified accurately by nutritionists.

## Introduction

Visual impairment is a disorder in the visual system characterized by partial or total loss of vision, which cannot be fixed with glasses, contact lenses, medication, or surgery [1]. Low vision occurs when the visual acuity is between 0.3 and 0.05 in the best eye [1] or when its visual field is inferior to 20° [2]. Blindness occurs when visual acuity is equal to or less than 0.05 [1] or the visual field is inferior to 10° [2].

Visually impaired people (VIP) have several difficulties related to diets, comprising the acquisition, preparation, and consumption of meals, which may certainly influence food choices [3, 4]. Living alone; low motivation; fear of domestic accidents, such as cuts and burns; and the convenience of eating out are some of the various obstacles frequently reported [5].

The greater the severity of the visual impairment, the worse the quality of life of VIP [6–8]. Besides feeding difficulties, Chronic Non-Communicable Diseases (e.g. diabetes, and hypertension), overweight due to inadequate nutrition, and other risk factors for cardiovascular diseases are also highly prevalent among these subjects [3].

This reality highlights the need to periodically monitor the nutritional status and dietary intake of VIP. Besides mitigating the aforementioned problems, studies indicate that a balanced and diversified diet also contributes to the prevention or reduction of the progression of the main causes of visual impairment, such as age-related macular degeneration (AMD) and diabetic retinopathy, since both have increased oxidative stress in the retina in common [9].

However, traditional methods for dietary intake assessment are not suitable for VIP. Dietary records, diet history, 24-hour dietary recalls, and food frequency questionnaires depend on the subjects ability to describe and quantify food consumption [10–14], which is hardly feasible for VIP because of their low active role in their diets [15]. Generally, their meals are prepared and/or portioned by others (e.g., family, cook, waiter, bartender, attendant) making it difficult for VIP to identify foods and report their quantities and characteristics accurately to health professionals [4]. The reduced handling of food in everyday life may also impair the memory related to the diet and compromise the responses to the dietary intake assessment methods [15]. Also, many VIP use exclusively the Braille system for written communication, resulting in incomprehensible dietary records for sighted health professionals [15].

It is important to highlight that asking others to report the food consumption of VIP on their behalf is not appropriate and often not possible. Many VIP live alone and may have no one to accompany them to appointments with nutritionists and report their food intake, while others are independent to conduct their daily activities with no companions or caregivers and there is no one to observe their food consumption throughout the day. Furthermore, requesting third parties to report food intake can lead to additional errors in the dietary assessment (e.g., inadequate description of foods and ingredients, underestimation or overestimation of portion sizes), especially if they have low education and/or do not have a close relationship with the VIP, as employees of establishments that serve food. People who eat in several places throughout the day would be the most affected, as there would be a great heterogeneity in the reports resulting from the subjective view of each companion or caregiver of VIP, regarding foods and their quantities.

Even when there is a person able to adequately report the food consumption of VIP, this can be interpreted as an undesirable attitudinal and communicational barrier [16] for those with this disability, because it hinders their guaranteed right to autonomy and protagonism in taking care of their health. In this context, considering the VIP as incapable can be understood as ableism, which must be fought through the use of different strategies that guarantee equity in health services.

Taking digital photographs of meals could be a way of overcoming these limitations, standing out as an attractive alternative to recording the daily food consumption of people with difficulties in reporting their diet [17], such as VIP. Its use proved to be precise for assessing energy and nutrient consumption in different settings, being comparable to doubly labeled water [18], and food records [18–20]. Also, the quantification of food portions in this method is comparable to that obtained by the weighed food records, with the advantage of being quick and minimizing changes in the report, since extensive methods reduce the engagement of the individual and worsen the quality and veracity of the information [21, 22].

Using mobile technologies, such as smartphones, has been indicated for photographing meals since traditional cameras are not practical and agile in collecting information in daily life [21]. This strategy has been validated for sighted individuals [18–22], but is also feasible for VIP, since many of them use smartphones with the support of accessibility tools [23, 24].

Protocols for VIP to autonomously conduct food records based on photographic images (FR-PI) were recently developed and validated [24], so that these subjects could satisfactorily photograph their daily meals using a smartphone. Besides image quality, these protocols were developed prioritizing the autonomy, safety, and discretion of the subjects, using the smartphone in two different positions, with its camera facing the meal or above it. After learning the protocol step-by-step with the help of health professionals, VIP can photograph their daily meals autonomously in different settings. This strategy was recognized by the authors as feasible to qualitatively assess VIP dietary intake, with the potential to be used both individually in nutritional assistance and epidemiological studies [24].

Studies on the food consumption and dietary intake of VIP are still scarce, especially regarding the FR-PI, outlined as a promising alternative to improve nutritional care in this population, as this method favors their autonomy, protagonism, and inclusion in health services.

We hypothesize that the FR-PI could be a useful tool for nutritionists in clinical settings to assess the dietary intake of VIP. Thus, we aim to investigate whether it is possible for nutritionists to accurately identify foods and their quantities using food records based on photographic images captured with a smartphone by visually impaired people.

## Materials and methods

### Study design, location, and population

This is an observational cross-sectional study, conducted with a panel of experts composed of nutritionists (n = 20) experienced in assessing food consumption over the last five years. All nutritionists selected had to meet two or more of the following requirements: 1) being a health system worker who routinely assesses the food consumption of patients; 2) being experienced in using a food atlas of portions/preparations in food surveys or clinical practice; 3) being responsible for research involving food consumption and dietary intake; 4) being a university professor responsible for teaching dietary intake assessment.

A non-probabilistic intentional sampling was adopted to select the nutritionists of the panel, aimed at including highly qualified Brazilian professionals with experience in the studied subject. Their recruitment was conducted by email. Those with irregular access to computers and the internet were considered ineligible since the panel was held only by digital means.

People with blindness and low vision [25] have also participated in the study. They were from a philanthropic institution for the rehabilitation of VIP and a federal university in the capital of the state of Rio Grande do Norte, Brazil.

As a large sample size is hard to reach in studies conducted with people with vision disorders [26], Respondent Driven Sampling was used to select these participants [26, 27]. A minimum sample size of 30 VIP participants was initially defined for this study, considering that when the degree of freedom increase (n-1) surpasses 30, the 95% confidence limit of the “t” distribution is similar to the “z” distribution.

Acquaintances of enrolled participants with visual disorders were also invited to participate in the study, through a face-to-face meeting, or by email, telephone, or social network application. VIP of all sexes aged 16 and over were considered eligible. We have excluded those with other types of disabilities or health problems that hinder the proper use of smartphones, and those with difficulty understanding and executing the procedures instructed by the researchers (e.g., amputation, paresis or paralysis of upper limbs, hearing loss, mental disorders).

### Data collection

All participants took part in a brief interview, conducted by the researchers, regarding socioeconomic, demographic, and health aspects.

The panel of experts received an online form containing images of three standardized meals, photographed by the VIP with a smartphone in two different positions, facing the meal, with the camera at 45° (Frontal Photo); and above the meal, with the camera at 90° (Aerial Photo). Details on the protocol adopted are available in the validation study by Borges et al. [24].

The three standardized meals were prepared by the researchers based on the most consumed foods in Brazil [28], representing the main daily meals for Brazilians, which are breakfast (coffee with milk, bread with cheese, banana), lunch/dinner (rice, beans, chicken steak, lettuce, tomatoes, carrots, natural juice), and snacks (cake, processed juice). Solid foods were served on a white dinner plate, and to better identify the items through photographs, their portioning did not overlap.

Researchers conducted a weighed food record (FR-W) when portioning food for data collection for all meals photographed, using a digital scale with 1g precision (Balmak® model Nutri 5).

The VIP participants were asked to take three subsequent photos of each meal, and the best one regarding framing, focus, and angle according to the researchers was used. Then, from the photographs used, one image of each meal was randomly selected for both protocols, resulting in a set of six photos sent to the panel of experts, they were the breakfast Frontal Photo; breakfast Aerial Photo; lunch/dinner Frontal Photo; lunch/dinner Aerial Photo; snack Frontal Photo; snack Aerial Photo. The images were inserted randomly and on different pages of the online form so that nutritionists did not examine frontal and areal photos of the same meal at the same time. No digital corrections were made to the photos to improve their quality.

After careful observation of the images, the nutritionists were asked to report an estimated FR-PI, discriminating which foods were present in each meal, and providing their quantities separately (portion size; household measures; the number of portions; grams or milliliters). All nutritionists analyzed the same set of photos in a single round and produced six FR-PI. There was no loss of information at this stage of the study.

### Statistical analysis

The FR-PI produced by the panel of experts was compared to the FR-W. The frequencies of the correctness of the experts concerning the number of food items and which were the ones present in the meal were determined from the comparison of the foods listed in the FR-W and those mentioned in the FR-PI.

To determine this correctness concerning the amount of food in the meal, the quantities of all food items described by the experts in the FR-PI were converted into grams or milliliters and compared to the real one, measured by the FR-W. Correctness was defined when the amount reported by the expert in the FR-PI was within a tolerance margin of 10% for more or less in relation to the value measured by the FR-W.

A statistically significant difference between the frequencies of correctness for the frontal and aerial photos was assumed when there was no overlap of their 95% confidence intervals, calculated by the software EpiInfo 3.5.4.

The Wilcoxon test was used to compare the absolute amount of food (grams or milliliters) and nutrients assessed by FR-W and FR-PI for both protocols, adopting 5% as statistical significance, using the Statistical Package for the Social Sciences software, version 23.0.

Due to ethical restrictions, data cannot be shared publicly, as participants would be easily identified with the variables listed. Data are available from the research ethics committee of Hospital Universitário Onofre Lopes (contact via cep.huol@ebserh.gov.br) for researchers who meet the criteria for access to confidential data.

### Ethics

This study was approved by the Research Ethics Committee of the Onofre Lopes University Hospital of the Federal University of Rio Grande do Norte (opinion 2.932.978; CAAE: 97423318.7.0000.5292) and followed all the ethical principles of the Declaration of Helsinki and the Resolution 466/2012 of the Brazilian National Health Council. Written informed consent was obtained from all subjects before taking part in the study, including from parents or guardians of the minors.

## Results

The nutritionists participating in the panel of experts were all female, and most were aged ≥40 years (55%) and with ≥10 years of working experience in dietary intake assessment (85%). Although a graduate degree was not an eligibility criterion, most had a Ph.D. in nutrition or related areas (60%).

Most VIP enrolled (n = 40) were male (67.5%), aged ≥ 40 years (57.5%; Min-Max: 16–70 years), non-white (60%), with complete high school or college degree (65%), and received ≥0.5 minimum wage of family income per capita (62.5%). There was a predominance of participants with blindness (77.5%) and without other disabilities besides the visual one (95%). Their use of smartphones was habitual (72.5%), and although only half used to take pictures using the cell phone camera, most (82.5%) reported not having any difficulties in making the photographic records based on the instructions provided.

For both frontal and aerial photos, when FR-PI were compared to the FR-W, the frequency of correctness for the number of food items present in the meal was 95.0% for breakfast, 100% for lunch/dinner, and 100% for the snack.

Concerning which were the food items present in the meals, all experts could identify 11 out of 13 (84,7%). For both protocols, some experts faced difficulties to recognize coffee with milk and grilled chicken fillet from the images captured (Table 1).

**Table 1:** Experts’ correctness in the evaluation of the meals. Natal, Rio Grande do Norte, Brazil -2019.

Food items	Item presence in the meal				Item amount in the meal			
	FRONTAL PHOTO		AERIAL PHOTO		FRONTAL PHOTO		AERIAL PHOTO	
	% ^a^	IC 95% ^b^	% ^a^	IC 95% ^b^	% ^c^	IC 95% ^b^	% ^c^	IC 95% ^b^
White bread	100.0	100–100	100.0	100–100	100.0	100–100	95.0	75.1–99.9
Sliced cheese	100.0	100–100	100.0	100–100	30.0	11.9–54.3	30.0	11.9–54.3
Coffee with milk	90.0	68.3–98.8	90.0	68.3–98.8	80.0	56.3–94.3	75.0	50.9–91.3
Banana	100.0	100–100	100.0	100–100	0.0	-	0.0	-
White rice	100.0	100–100	100.0	100–100	35.0	15.4–59.2	35.0	15.4–59.2
Beans	100.0	100–100	100.0	100–100	0.0	-	0.0	-
Grilled chicken fillet	90.0	68.3–98.8	85.0	62.1–96.8	20.0	5.7–43.7	20.0	5.7–43.7
Grated carrot	100.0	100–100	100.0	100–100	5.0	0.1–24.9	5.0	0.1–24.9
Lettuce	100.0	100–100	100.0	100–100	15.0	3.2–37.9	15.0	3.2–37.9
Sliced tomato	100.0	100–100	100.0	100–100	35.0	15.4–59.2	45.0	23.1–68.5
Orange juice	100.0	100–100	100.0	100–100	60.0	36.1–80.9	55.0	31.5–76.9
Cake	100.0	100–100	100.0	100–100	5.0	0.1–24.9	0.0	-
Boxed grape juice	100.0	100–100	100.0	100–100	100.0	100–100	100.0	100–100

^a^ Frequency (%) of experts reporting the food item

^b^ 95% confidence interval (IC95%)

^c^ Frequency (%) of experts reporting amount within a tolerance margin of 10% more or less in relation to the weighed food record

On the other hand, only two of the food items (15,3%) had their amount correctly estimated by all experts: white bread and boxed grape juice. Bananas, beans, grated carrots, and cakes were the foods with the lowest correctness, regardless of the position in which the photograph was taken (Table 1).

The amount of most of the food items based on the frontal photos (7 items, 53.8%), and on the aerial photos (8 items, 61.5%) was significantly underestimated in FR-PI. For both protocols, the amount of chicken was significantly overestimated, while for drinks it did not differ between FR-PI and FR-W (Table 2).

**Table 2:** Amount of food present in the meal, according to the weighed food record (FR-W) and the estimated food record based on photographic images (FR-PI) captured by the visually impaired participants. Natal, Rio Grande do Norte, Brazil -2019.

Food items	FR-W	FR-PI					
		FRONTAL PHOTO			AERIAL PHOTO		
	Amount	Amount^a^	Δ	p-value^b^	Amount^a^	Δ	p-value^b^
	(g/ml)	(g/ml)	(g/ml)		(g/ml)	(g/ml)	
White bread	55.1	50.0	-5.1	< 0.000	50.0	-5.1	< 0.000
Sliced cheese	42.2	22.7	-19.5	< 0.000	21.7	-20.5	< 0.000
Coffee with milk	200.0	181.5	-18.5	0.066	177.5	-22.5	0.059
Banana	112.1	86.0	-26.1	< 0.000	83.2	-28.9	< 0.000
White rice	83.6	66.7	-16.9	0.024	65.6	-18.0	0.011
Beans	101.6	84.9	-16.7	0.002	82.9	-18.7	0.002
Grilled chicken fillet	81.2	124.5	+43.3	0.001	123.5	+42.3	0.002
Grated carrot	30.8	18.7	-12.1	< 0.000	18.7	-12.1	< 0.000
Lettuce	16.8	14.5	-2.3	0.063	15.0	-1.8	0.016
Sliced tomato	34.0	37.3	+3.3	0.056	40.7	+6.7	0.001
Orange juice	200.0	191.7	-8.3	0.369	184.2	-15.8	0.129
Cake	70.7	57.5	-13.2	< 0.000	57.5	-13.2	< 0.000
Boxed grape juice	200.0	200.0	0.0	1.000	200.0	0.0	1.000

Δ: Mean difference between the amount assessed by FR-PI and FR-W

^a^ Mean of the amount values reported by all experts

^b^ Comparison of the amounts assessed by FR-PI and FR-W using the Wilcoxon test

When comparing the quantity of nutrients obtained by FR-W and FR-PI, there was a significant underestimation of energy, carbohydrate, protein, lipid, fiber, calcium, iron, and vitamin C for half of the food items in both protocols (6 items, 46,2%). Overestimation of nutrients was rarely observed for frontal photos (1 item, 7,7%) and aerial photos (2 items, 15,4%). The quantity of nutrients correctly estimated for both frontal photos (5 items, 38,5%) and aerial photos (30,8%) was low (Table 3).

**Table 3:** Quantity of nutrients present in the meal, according to the weighed food record (FR-W) and the estimated food record based on photographic images (FR-PI) captured in frontal photos by the visually impaired participants. Natal, Rio Grande do Norte, Brazil -2019.

Food items	Energy		Carbohydrate		Protein		Lipid		Fiber		Calcium		Iron		Vitamin C	
	(kcal)		(g)		(g)		(g)		(g)		(mg)		(mg)		(mg)	
	FR-W	FR-PI^a^	FR-W	FR-PI^a^	FR-W	FR-PI^a^	FR-W	FR-PI^a^	FR-W	FR-PI^a^	FR-W	FR-PI^a^	FR-W	FR-PI^a^	FR-W	FR-PI^a^
	FRONTAL PHOTO															
White bread	164,82	149,5^b^	33,95	30,8^b^	5,41	4,92^b^	1,16	1,06^b^	1,44	1,31^b^	7,83	7,10^b^	2,39	2,17^b^	0,00	0,00
Sliced cheese	145,76	78,49^b^	0,08	0,05^b^	10,22	5,51^b^	11,66	6,28 ^b^	0,00	0,00	338,85	182,46^b^	0,11	0,06^b^	0,00	0,00
Coffee with milk	95,60	86,76	9,29	8,44	4,50	4,09	4,69	4,26	0,00	0,00	151,70	137,7	0,10	0,09	0,00	0,00
Banana	118,85	91,16^b^	29,04	22,27^b^	1,24	0,95^b^	0,31	0,24^b^	2,19	1,68^b^	6,93	5,31^b^	0,43	0,33^b^	24,22	18,57^b^
White rice	114,56	91,45^b^	24,33	19,4^b^	2,00	1,60^b^	1,32	1,06^b^	1,06	0,85^b^	4,88	3,89^b^	0,28	0,22^b^	0,38	0,30^b^
Beans	71,13	59,43^b^	15,54	12,99^b^	4,84	4,05^b^	0,54	0,46^b^	7,17	5,99^b^	29,17	24,37^b^	1,41	1,18^b^	0,00	0,00
Grilled chicken **fillet**	121,87	186,8^b^	0,00	0,00	26,00	39,8^b^	2,01	3,09^b^	0,00	0,00	4,34	6,65^b^	0,27	0,41^b^	0,00	0,00
Grated carrot	9,26	5,61^b^	2,33	1,41^b^	0,34	0,21^b^	0,06	0,04^b^	0,92	0,56^b^	6,61	4,00^b^	0,15	0,09^b^	1,58	0,95^b^
Lettuce	1,51	1,31	0,22	0,19	0,28	0,25	0,02	0,02	0,31	0,27	6,41	5,51	0,07	0,06	2,63	2,26
Sliced tomato	5,7	6,33	1,29	1,42	0,35	0,39	0,05	0,06	0,54	0,60	2,36	2,59	0,10	0,11	5,27	5,77
Orange juice	74,00	70,9	16,9	16,24	1,38	1,32	0,22	0,21	0,34	0,01	15,06	14,44	0,04	0,00	139,4	133,63
Cake	309,17	251,3^b^	39,47	32,09^b^	5,12	4,17^b^	14,71	11,96^b^	0,59	0,48^b^	29,3	23,81^b^	1,67	1,36^b^	0,00	0,00
Boxed grape juice	51,00	51,0	12,00	12,0	0,00	0,00	0,00	0,00	0,00	0,00	0,00	0,00	0,00	0,00	30,00	30,00
	AERIAL PHOTO															
White bread	164,82	149,5^b^	33,95	30,8^b^	5,41	4,92^b^	1,16	1,06^b^	1,44	1,31^b^	7,83	7,10^b^	2,39	2,17^b^	0,00	0,00
Sliced cheese	145,76	75,04^b^	0,08	0,04^b^	10,22	5,26^b^	11,66	6,00^b^	0,00	0,00^b^	338,85	174,4^b^	0,11	0,06^b^	0,00	0,00
Coffee with milk	95,60	84,8	9,29	8,25	4,50	4,00	4,69	4,16	0,00	0,00	151,70	134,63	0,10	0,09	0,00	0,00
Banana	118,85	88,19^b^	29,04	21,55^b^	1,24	0,92^b^	0,31	0,23^b^	2,19	1,62^b^	6,93	5,14^b^	0,43	0,32^b^	24,22	17,97^b^
White rice	114,56	89,91^b^	24,33	19,1^b^	2,00	1,58^b^	1,32	1,04^b^	1,06	0,83^b^	4,88	3,83^b^	0,28	0,22^b^	0,38	0,29^b^
Beans	71,13	58,0^b^	15,54	12,68^b^	4,84	3,95^b^	0,54	0,45^b^	7,17	5,85^b^	29,17	23,79^b^	1,41	1,15^b^	0,00	0,00
Grilled chicken **fillet**	121,87	185,3^b^	0,00	0,00	26,00	39,5^b^	2,01	3,06^b^	0,00	0,00	4,34	6,59^b^	0,27	0,41^b^	0,00	0,00
Grated carrot	9,26	5,61^b^	2,33	1,41^b^	0,34	0,21^b^	0,06	0,04^b^	0,92	0,56^b^	6,61	4,00^b^	0,15	0,09^b^	1,58	0,95^b^
Lettuce	1,51	1,35	0,22	0,20	0,28	0,26	0,02	0,02	0,31	0,27	6,41	5,70	0,07	0,06	2,63	2,34
Sliced tomato	5,7	6,93^b^	1,29	1,56^b^	0,35	0,42^b^	0,05	0,07^b^	0,54	0,65^b^	2,36	2,83^b^	0,10	0,12^b^	5,27	6,31^b^
Orange juice	74,00	68,2	16,9	15,60	1,38	1,27	0,22	0,20	0,34	0,31	15,06	13,87	0,04	0,04	139,4	128,40
Cake	309,17	251,3^b^	39,47	32,09^b^	5,12	4,17^b^	14,71	11,96^b^	0,59	0,48^b^	29,3	23,81^b^	1,67	1,36^b^	0,00	0,00
Boxed grape juice	51,00	51,0	12,00	12,0	0,00	0,00	0,00	0,00	0,00	0,00	0,00	0,00	0,00	0,00	30,00	30,00

^a^ Mean of quantity values reported by all experts

^b^ p-value <0.05 for the comparison between the amount assessed by FR-PI and FR-W

## Discussion

This unprecedented study has demonstrated that health professionals can identify food items in meals photographed by VIP, both with frontal and aerial photos. This finding has great value since the qualitative assessment of dietary intake is indispensable for knowing the eating habits of individuals, as well as to seek the most appropriate strategies to promote a healthy diet.

Understanding the various combinations of foods and their forms of preparation, as well as the social and cultural influence on food, is as important as measuring nutrient intake [29]. In fact, knowing the type of food present in daily meals has gained increasing prominence, since the level of processing and other aspects of the food products affect the quality of diets and modify the risk for obesity and several chronic non-communicable diseases [30, 31].

Although the quality and accuracy of dietary information are extremely pursued [17], for various segments of the population, the use of traditional methods of assessing food consumption is bound to error, especially in low-resource settings. As an alternative, innovative technologies that are less demanding and more suitable for specific populations have been increasingly studied to obtain accurate and reliable dietary information, as is the case of mobile phones. Defined as an image-assisted dietary assessment method, the use of photographs captured by mobile phones during eating episodes enhances accuracy, reduces respondent burden, and is easy to use [17].

This method has also revealed great potential for health professionals to know what is being consumed by VIP throughout the day when using protocols created specifically for the characteristics of this population [24], as confirmed by this study. So far, the FR-PI is the only dietary assessment tool specifically adapted for VIP, and by providing step-by-step instructions to ensure proper positioning of the body to capture photographs at the correct angles [24], it stands out from other image-based methods, eliminating the need for a non-disabled person. Thus, the use of FR-PI may be considered a breakthrough in nutritional assistance for VIP since it overcomes the predominant biological perspective of dietary assessment, ensuring autonomy and privacy of the individual, promoting inclusion in the health system, and safeguarding ​​equity.

It is undeniable that, in this study, professionals had difficulties identifying some food items, such as grilled chicken fillets, which were mistaken for breaded chicken fillets or meat steaks. Orange juice also had its flavor mistakenly reported, which was expected even with the use of a transparent glass cup, as it is very difficult to determine flavor just by the color.

However, this could be easily overcome by including an audio description of the meal, sent by the VIP to the health professional along with the images, thus complementing the dietary information [15]. Additional descriptions of meals, in addition to the photos, have been pointed out as relevant in studies with sighted individuals using this method, to provide more accurate dietary data [18–22, 32–35]. Thus, besides the flavor of the foods and drinks, it would be possible to collect further information on the cooking method, hidden ingredients and foods, and added condiments [17].

Contrastingly, in this study, the FR-PI captured by VIP has not been proven to be accurate to assess the quantity of food and nutrients in the meals, which could be a limitation of its use in numerous types of epidemiological, clinical, or basic research studies that require these details. Only for a few items, those whose portion is generally standardized and widely known, the amount was correctly estimated by most of the experts, which hindered the quantitative assessment of nutrients of the meal. The correctness of the experts regarding the quantity of the boxed grape juice was already predicted, as it was served in its original packing with details on quantity per unit and nutritional facts.

Christoph et al. [32] found consistent results both for the identification of food groups and for the estimation of the number of portions from the use of digital photography captured by sighted individuals. However, as this study evaluated only the absolute amount of food in grams or milliliters, further studies need to be conducted to assess whether the number of food portions would be correctly identified based on the photographs taken by the VIP.

It is important to highlight that estimating food amounts based on photographs is quite difficult, and this is not always related to the image quality, as verified in this study, in which the pictures presented enough overall quality to allow the identification of all foods by almost all experts. For this reason, some strategies have been recommended to improve FR-PI, such as comparing the photographs with images of reference portions for known food quantities [36–38], and requiring that the subjects place a fiducial marker (e.g., fork, pen, coin) close to the food items before taking the pictures to facilitate the estimation [17]. Nevertheless, aiming to simulate everyday situations in low-resource settings, this study did not impose on the professionals the method for estimating the amount of food, nor included a standard-sized fiducial marker beside the meals, as in real settings its use is highly unlikely (e.g., eating popcorn at the movies, having a snack on a school bench, having dinner on a date).

It is also reported that the standardization of the angle of the photo and the distance from the camera are important to identify the size and number of portions, by understanding the depth and height of the food items in the dish [39, 40]. However, in this study, there was no difference between the estimated food amount when the camera was positioned facing the meal, at 45°, or above it, at 90°.

It is worth noting that the use of videos instead of photographic images to assess diet has not yet been sufficiently tested in the literature, nor does it seem to be a good alternative to overcome the limitations of the FR-PI based on images captured by VIP. Small tremors when recording short and/or low-resolution videos can make it difficult to identify foods and their quantities. In addition, long and/or high-resolution videos demand more memory space on smartphones, which could interfere with the exchange of files between the VIP and the nutritionist. Furthermore, the professional may face difficulties to pause the video at an exact moment, or enlarging the images (zoom function).

As this was the first study to test whether nutritionists were able to accurately identify foods and their amounts based on photographic images captured by VIP using a smartphone, it was conducted under controlled conditions, in which food items did not overlap in the plate, and standardized meals with low complexity dishes were used. Also, to minimize the fatigue of the experts, a limited number of photographic images were used.

However, considering the potential of the method according to the results, it is now necessary to proceed with further investigation of its use. As stated by Christoph et al. [32], the use of digital photography must be confirmed in conditions that simulate people’s daily lives, including food portioning with different utensils, complex recipes, and several ingredients; besides arranging different meal items on the plate the way it is commonly made at home or served in restaurants and other food establishments. Also, the correct use of the protocol needs to be evaluated in VIP in free-living conditions.

Still, this innovative and pioneering study was developed with a panel of experts with a far superior number of members than any other international research on FR-PI [37, 41–43], and with tolerable sample size, considering the difficulty to access and enroll a large number free-living disabled people in studies similar to this one. Our results stand out amid the scarcity of scientific literature related to the dietary intake of VIP, and reveals that the FR-PI captured by these subjects can be useful to enable a qualitative assessment of their diets in clinical and epidemiological settings and to guide the often-neglected nutritional help by health professionals.

Also, the FR-PI protocol for VIP is easy to be learned and memorized with the help of health professionals, so that these subjects can photograph their daily meals in different settings autonomously. By using this easy-to-perform and low-cost method, it is possible to reveal, in the short term, the quality of the diet and which foods are consumed in specific meals. In the long term, it may bring to light inappropriate eating patterns, such as the regular consumption of processed and ultra-processed foods, low frequency of nutrient food sources, unbalanced meals, and undesirable food combinations. Adherence to international food-based dietary guidelines [29] is an ascendant approach that can also be accessed with this method, providing rich information on the risk for malnutrition and chronic non-communicable diseases that can guide the development of actions to promote healthy eating among VIP [44].

## Conclusion

Some food items had significant differences statistically compaired to the weighed food record, denoting that the photographic images may not suitable for the quantitative assessment of food consumption. However, most foods could be correctly identified by the nutritionists using the photographic images captured by visually impaired people. Besides, the amount of many food items were relatively close to that identified by the weighed food record, and these errors were similar to what would be expected in traditional dietary assessment methods based on recall or estimations (e. g. estimated food record, 24-hour dietary recalls).

Thus, food records based on photographic images captured by visually impaired people proved to be a promising tool to improve nutritional care in this population and to promote their autonomy, protagonism, and inclusion in public health services.

Further and more robust studies must confirm, in free-living conditions, the accuracy and the social impact of this mobile-based technology for collecting dietary data of visually impaired people.
